# Activation of cyclin D1-related kinase in human lung adenocarcinoma

**DOI:** 10.1038/sj.bjc.6690752

**Published:** 1999-10

**Authors:** K Igarashi, T Masaki, Y Shiratori, W Rengifo, T Nagata, K Hara, T Oka, J Nakajima, T Hisada, E Hata, M Omata

**Affiliations:** Second Department of Internal Medicine, Department of Pathology, Department of Cardiothoracic Surgery, Faculty of Medicine, University of Tokyo, 7-3-1 Hongo Bunkyo-ku, 113, Japan; Division of Respiratory Medicine, Tokyo Teishin Hospital, Chiyoda-ku, Tokyo, 102, Japan; Surgical Department of Respiratory Center, Mitsui Memorial Hospital, Chiyoda-ku, Tokyo, 101, Japan

**Keywords:** cyclin D, cyclin-dependent kinase, kinase activity, Rb protein, lung cancer

## Abstract

Cyclin D1 gene amplification is an important event in many cancers, but it is rarely found in non-small-cell lung cancer (NSCLC). This study was conducted in an attempt to clarify any other mechanisms related to cyclin D1 involvement in the malignant transformation of NSCLC, and we clearly showed for the first time that cyclin D1-related kinases are activated in NSCLC, especially in adenocarcinoma but not in squamous cell carcinoma. The results of this study strongly suggest that enhanced cyclin D1-related kinase activity could contribute to a progression of adenocarcinoma in NSCLC. © 1999 Cancer Research Campaign


*British Journal of Cancer* (1999) **81**(4), 705–711
© 1999 Cancer Research Campaign
Article no. bjoc.1999.0752

### Activation of cyclin D1-related kinase in human lung adenocarcinoma

**K Igarashi[1], T Masaki[1], Y Shiratori[1], W Rengifo[1], T Nagata[1], K Hara[2], T Oka[2], J Nakajima[3], T Hisada[4], E Hata[5] and M Omata[1]**

[1]Second Department of Internal Medicine, [2]Department of Pathology and [3]Department of Cardiothoracic Surgery, Faculty of Medicine, University of Tokyo, 7-3-1 Hongo, Bunkyo-ku, Tokyo 113, Japan; [4]Division of Respiratory Medicine, Tokyo Teishin Hospital, Chiyoda-ku, Tokyo 102, Japan; [5]Surgical Department of Respiratory Center, Mitsui Memorial Hospital, Chiyoda-ku, Tokyo 101, Japan

**Summary** Cyclin D1 gene amplification is an important event in many cancers, but it is rarely found in non-small-cell lung cancer (NSCLC). This study was conducted in an attempt to clarify any other mechanisms related to cyclin D1 involvement in the malignant transformation of NSCLC, and we clearly showed for the first time that cyclin D1-related kinases are activated in NSCLC, especially in adenocarcinoma but not in squamous cell carcinoma. The results of this study strongly suggest that enhanced cyclin D1-related kinase activity could contribute to a progression of adenocarcinoma in NSCLC. © 1999 Cancer Research Campaign

**Keywords:** cyclin D; cyclin-dependent kinase; kinase activity; Rb protein; lung cancer

Progression of cells through the cell cycle is governed by the sequential formation and degradation of a series of cyclins that complex with and activate several cyclin-dependent kinases (cdk) (Okayama et al, 1996). There are at least 11 distinct cyclin genes in the human genome which can be categorized as follows: G1 phase cyclins (C, D1–3, E, G and H), S phase cyclins (A and F) and G2/M phase cyclins (A and B1–2) (Hunter and Pines, 1994). Among them, the human cyclin D1 gene was first cloned through its ability to complement a *Saccharomyces cerevisiae* strain that had mutations in all three of the known yeast G1 cyclins (Matsushime et al, 1991). Immunoneutralization with cyclin D1 antibodies resulted in cell cycle arrest in the G1 phase (Baldin et al, 1993), and cyclin D1 overexpression shortens the G1 phase of the cell cycle in cultured cells, decreases cell size, and makes cells less dependent on exogenous growth factors (Quelle et al, 1993). In addition, cyclin D1 stimulates progression through the G1 phase in association with its catalytic partners cdk4 and cdk6. Cyclin D1-related kinase phosphorylates the retinoblastoma protein (pRb) and released transcriptional factors (collectively termed E2Fs) enhance cell cycle activity (Taya, 1995). Therefore, the cyclin and cdk complexes may play a critical role in cell proliferation and differentiation (Sherr, 1996).

It has been reported that gene amplification of cyclin D1 occurs in many tumour cells (Hall and Peters, 1996). Although cyclin D1 gene amplification is an important event in many human cancers, but is rarely found in lung cancer (Berenson et al, 1990; Betticher et al, 1996). On the other hand, the activity levels of cyclin D1-related kinase in lung cancer has yet to be clarified. We now report for the first time that cyclin D1-related kinase, the protein level of cyclin D1, and its catalytic subunit cdk4 and cdk6 are elevated in lung cancers, especially in adenocarcinoma.

*Received 7 October 1998*
*Revised 18 March 1999*
*Accepted 20 April 1999*

*Correspondence to:* K Igarashi

### MATERIALS AND METHODS

### Human tissues

Tumour and resection margin samples were obtained by surgery from 26 patients (21 males and five females, mean age 62.1; range 41–80 years). The clinical backgrounds and characteristics of the patients are shown in Table 1. None of the patients had received chemo- or radiation therapy before surgery. Among them, 11 were in stage I, three in stage II, nine in stage IIIA, one in stage IIIB and two in stage IV (UICC, 1987). Adenocarcinoma and squamous cell carcinoma were found in 16 and ten cases respectively, according to the standard WHO criteria (1981). In each case, the resected lung lobe was divided into tumourous (T) and non-tumourous (N) regions visually, which were histologically confirmed. All preparations were stored at –80°C until required for experiments.

### Chemicals

Chemicals were purchased from Sigma Chemical Co. (St Louis, MO, USA) or Wako Pure Chemical Co. (Tokyo, Japan). The monoclonal antibody against cyclin D1 (HD-11) was obtained from Santa Cruz Biotechnology Inc. (Santa Cruz, CA, USA). This antibody reacts with cyclin D1 of mouse, rat and human origin by Western blot and immunoprecipitation, and is non-cross-reactive with other cyclins (Meyerson and Harlow, 1994). Anti-cdk4 (H303), anti-cdk6 (C21), anti-Rb (C-15)-G and anti-E2F-1 (C-20) were also purchased from Santa Cruz Biotechnology Inc.. This antibody against pRb recognizes both phosphorylated and non-phosphorylated forms of Rb p110. On the other hand, the polyclonal antibody against phosphorylated pRb obtained from MBL Co. Ltd (Nagoya, Japan) reacts with only phosphorylated pRb at the site of Ser 780, and detects 105 kDa protein corresponding to amino acids 774–786 of human Rb. Cyclin D1–cdk4 specifically phosphorylates Ser 780 in pRb, but cyclin E–cdk2 and cyclin A–cdk2 does not phosphorylate its site (Kitagawa et al, 1996).

**Table 1** Clinicopathological features of the patients and T/N ratio of cyclin D1-related kinase activity

| Case | Age | Sex | Histology | Tumour size (cm) | Degree of differentiation | Clinical stage[a] | T/N ratio of kinase activity |
|---|---|---|---|---|---|---|---|
| 1 | 41 | male | Ad | 7 | Poor | I | 2.7 |
| 2 | 73 | male | Ad | 7 | Poor | IIIA | NT |
| 3 | 69 | male | Ad | 5 | Mod | IIIA | 4.7 |
| 4 | 51 | male | Ad | 5 | Poor | IIIA | 6.6 |
| 5 | 63 | male | Ad | 5 | Poor | IIIB | 2.5 |
| 6 | 54 | male | Ad | 4 | Mod | I | 6.4 |
| 7 | 55 | female | Ad | 4 | Mod | II | 2.7 |
| 8 | 63 | male | Ad | 4 | Mod | IV | NT |
| 9 | 49 | male | Ad | 3 | Mod | IV | 2.0 |
| 10 | 65 | male | Ad | 3 | Well | IIIA | 0.6 |
| 11 | 56 | female | Ad | 3 | Poor | IIIA | NT |
| 12 | 66 | male | Ad | 3 | Poor | IIIA | NT |
| 13 | 42 | male | Ad | 2.5 | Well | IIIA | NT |
| 14 | 76 | female | Ad | 2.5 | Well | I | NT |
| 15 | 62 | male | Ad | 2 | Mod | II | 1.1 |
| 16 | 59 | female | Ad | 1.5 | Well | I | 1.0 |
| 17 | 80 | male | Sq | 6 | Poor | II | 1.2 |
| 18 | 54 | male | Sq | 6 | Poor | IIIA | 1.5 |
| 19 | 62 | male | Sq | 6 | Mod | I | NT |
| 20 | 59 | male | Sq | 6 | Mod | I | NT |
| 21 | 78 | male | Sq | 4 | Mod | I | 0.1 |
| 22 | 62 | male | Sq | 4 | Poor | IIIA | 0.1 |
| 23 | 65 | male | Sq | 4 | Poor | I | NT |
| 24 | 68 | female | Sq | 2 | Mod | I | 1.3 |
| 25 | 72 | male | Sq | 2 | Mod | I | 1.3 |
| 26 | 70 | male | Sq | 1.5 | Mod | I | 0.1 |

[a]Clinical stages are classified using the UICC TNM classification system.

### Rb fusion protein

We used pRb protein expressed in *Escherichia coli* as a 46 kDa glutathione S-transferase (GST) fusion protein corresponding to amino acids 769–921 mapping within the carboxyterminal domain of pRb of mouse origin (Santa Cruz). This pRb was reported as an excellent substrate for cyclin D1/cdk4 or cdk6 (Matsushime et al, 1992; Meyerson and Harlow, 1994).

### Tissue lysates

The tissue samples were frozen using dry ice within 20 min of collection. The samples were homogenized in lysis buffer (50 mM N-2-hydroxyethylpiperazine-N′-2-ethanesulfonic acid (HEPES; pH 7.0), 250 mM sodium chloride (NaCl), 0.1% Nonidet P-40, 100 mM NaF, 200 μM sodium orthovanadate, 0.5 mM phenylmethylsulphonyl fluoride (PMSF), and 10 μg $ml^{-1}$ aprotinin) and lysates were centrifuged at 29 000 ***g*** for 20 min at 4°C twice. Protein concentration was measured by the bicin choninic acid protein assay.

### Gel electrophoresis and Western blot

Sodium dodecyl sulphate-polyacrylamide gel electrophoresis (SDS-PAGE) was performed according to the method of Laemmli (Laemmli, 1970), and Western blot was performed as described by Towbin et al (1979), using primary antibodies and horseradish peroxidase-linked secondary antibodies. Immunoreactive proteins were visualized with an enhanced chemiluminescence (ECL) detection system (Amersham) on X-ray film. The exposure times in the ECL method lasted for 30 s at room temperature for all samples. Cyclin D1 and cdk4 or cdk6 complexes were detected by immunoprecipitation (IP) with antibodies to cyclin D1, followed by immunoblotting with specific antibodies.

### Immunohistochemistry

Sections were immunohistologically stained according to the ABC method (Hsu et al, 1981). Resected lung specimens were immediately fixed in 4% paraformaldehyde for 4 h and embedded in paraffin. After conventional processing, sections (2-μm thick) were prepared, mounted on glass slides, and air-dried at room temperature overnight. The sections were then dewaxed in xylene and rehydrated in a series of graded alcohol. Phosphate-buffered saline (PBS; 10 mM) containing 0.5% hydrogen peroxide was used to block endogenous peroxidase activity. The sections were subsequently washed with PBS and immunostained by the ABC method. In brief, the sections were incubated with the primary antibody (cyclin D1 antibody, R-124, Santa Cruz) for 8 h, washed with PBS, incubated with the secondary antibody (horseradish-rabbit IgG anti-mouse immunoglobulin) for 2 h. Immunoreactivity was visualized using diaminobenzidine, and the sections were counterstained with Mayer's haematoxylin.

### Immunoprecipitations

The protein concentration of tissue lysates was adjusted to equivalent level (200 μg), and precleaned with immunobilized protein A (ImmunoPure: Pierce, Rockford, IL, USA). After incubation with anti-cyclin D1 or anti-Rb for 4 h on ice, they were precipitated with 50 μl of immunobilized protein A (50%, slurry). The samples were washed four times with IP buffer (50 mM HEPES (pH 8.0),

© 1999 Cancer Research Campaign

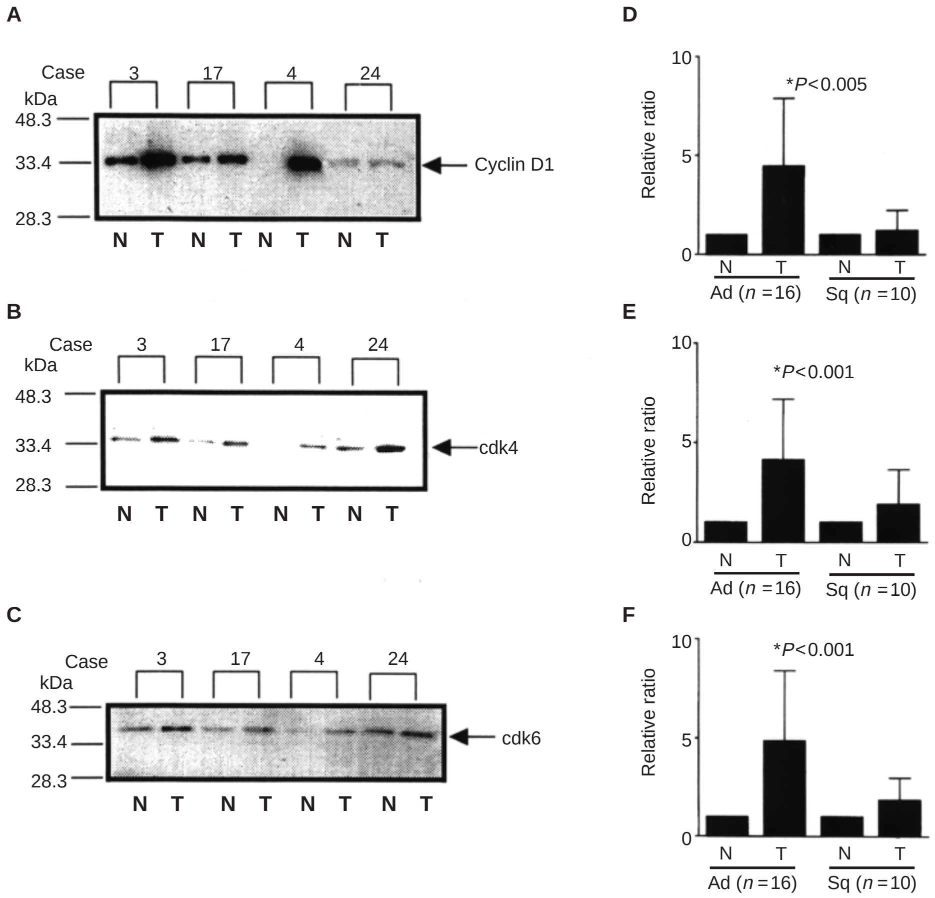

**Figure 1** Western blot of cyclin D1 (**A**), cdk4 (**B**) and cdk6 (**C**) using an enhanced chemiluminescence detection system. Sixty micrograms of human tissue lysate fraction of tumorous (T), and non-tumourous (N) regions of lung cancer were applied to SDS-PAGE and analysed as described in Methods. Cases 3 and 4; adenocarcinoma, cases 17 and 24; squamous cell carcinoma. (**D**, **E** and **F**) Densitometry of Western blot of cyclin D1, cdk4 and cdk6 in tumorous (T) and non-tumourous (N) portions of lung cancer. The relative ratio of the cyclin D1 (**D**), cdk4 (**E**) and cdk6 (**F**) band of tumorous tissues to the cyclin D1 (**D**), cdk4 (**E**) and cdk6 (**F**) band of the nontumorous regions of adenocarcinoma was 4.46 ± 3.40 ($P < 0.005$), 4.14 ± 3.01 ($P < 0.001$) and 4.84 ± 3.56 ($P < 0.001$) respectively

150 mM NaCl, 2.5 mM ethylene glycol-bis (β-aminoethyl ether)-*N*,*N*,*N*′,*N*′-tetraacetic acid (EGTA), 1 mM EDTA, 1 mM dithiothreitol (DTT), 0.1% Tween-20) containing 10% glycerol, 0.1 mM PMSF, 20 U of aprotinin per ml, 10 mM β-glycerophosphate, 1 mM NaF and 0.1 mM sodium orthovanadate, and once with 50 mM HEPES (pH 8.0) containing 1 mM DTT.

### In vitro kinase assays

Immunoprecipitates prepared with anti-cyclin D1 were suspended in 20 μl of kinase buffer (50 mM HEPES (pH 8.0), 10 mM magnesium chloride, 1 mM DTT, 2 mM glutathione (reduced form), 10 mM β-glycerophosphate, 1 mM NaF, 0.1 mM sodium orthovanadate, 20 μM ATP), and then incubated at 30°C for 30 min with 1 μg of GST-Rb fusion protein and 10 μCi of [$\gamma$-$^{32}$P] ATP (NEN Life Science Products Inc., Tokyo, Japan). After incubation, phosphoproteins were resolved by SDS-polyacrylamide (12.5%) gel electrophoresis (Matsushime et al, 1992). Phosphorylated proteins were visualized by autoradiography or image analysis by the BAS 2000 system (Fuji Photo Film Co. Ltd, Tokyo, Japan). The activity of cyclin D1-related kinase in each sample was obtained by averaging three measurements for each of the samples.

### Western blot and kinase activity

Density of the immunoreactive band for cyclin D1, cdk4 and cdk6 obtained on Western blot and density of the phosphorylated band of GST-Rb fusion protein obtained on autoradiography were analysed using an image analyser (Intelligent Quantifier I-D; Bio Image, Tokyo, Japan).

### Statistical analysis

Data are expressed as means ± S.D. The significance of differences between results was determined using the Student's *t*-test. A Spearman's rank-order correlation coefficient was calculated for correlation analysis.

### RESULTS

### Immunoblot of cyclin D1, cdk4 and cdk6 in lung cancer

The expression of cyclin D1, cdk4 and cdk6 were measured by Western blot analysis. Representative results on Western blot of cyclin D1, cdk4 and cdk6 are shown in Figure 1A, B and C respectively.

*© 1999 Cancer Research Campaign*

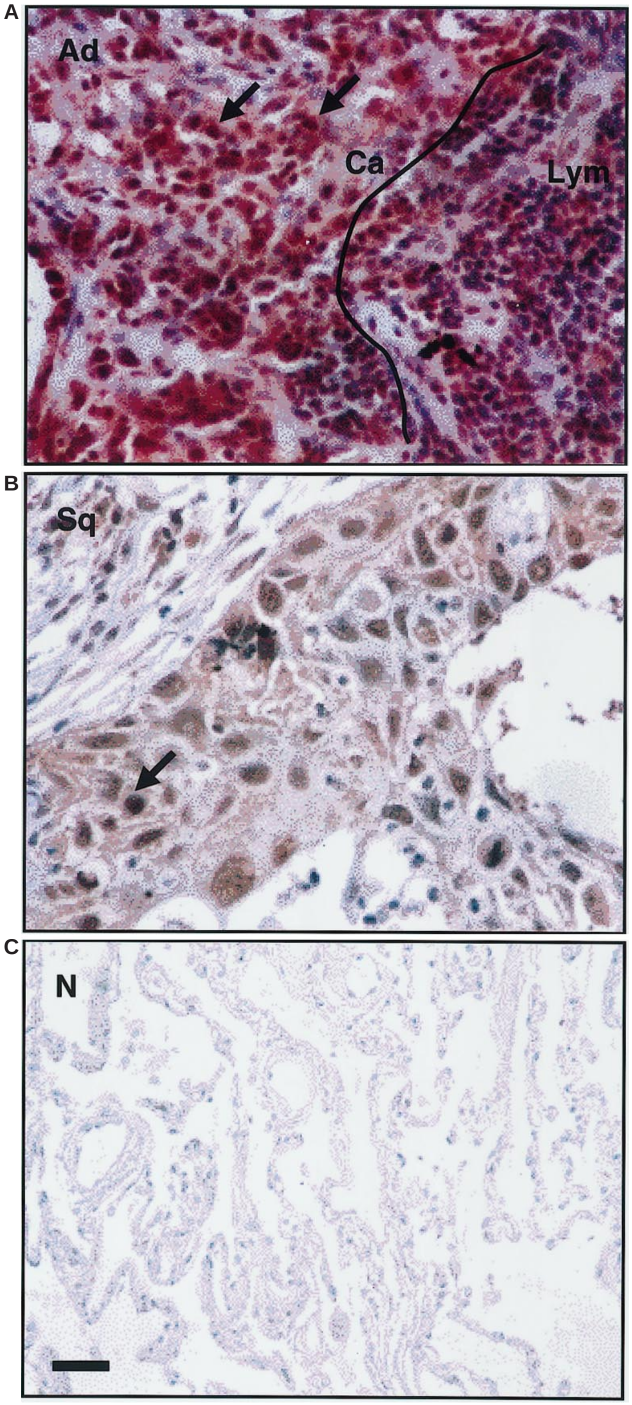

**Figure 2** Immunohistochemistry of cyclin D1 in the lung tissues of adenocarcinoma (**A**), squamous cell carcinoma (**B**) and corresponding normal tissue (**C**). In adenocarcinoma (Ad, case 3) with strong nuclear staining (arrows), the cytoplasm was also weakly stained, but it was rare to find staining in the infiltrating inflammatory cells (lymphocytes). In squamous cell carcinoma (Sq, case 21), cyclin D1 staining was weakly stained only in some cancer cells. Sections were counterstained with Mayer's haematoxylin. Labelling index in Ad and Sq was 80% and 20% respectively. Ca, cancerous areas; Lym, lymphocytes; bars = 100 μm

Each single band correspondent to 34, 34 and 38 kDa as a molecular size was cyclin D1, cdk4 and cdk6 respectively. The expression of cyclin D1, cdk4 and cdk6 proteins in lung cancer showed significantly higher levels than in adjacent non-cancerous regions, and the relative ratio of cyclin D1, cdk4 and cdk6 protein amounts were 3.10 ± 2.96 ($P < 0.005$), 3.32 ± 2.87 ($P < 0.001$) and 3.73 ± 3.31 ($P < 0.001$) respectively. When tumour cells were histologically classified, mean relative ratio of cyclin D1, cdk4 and cdk6 protein in adenocarcinomas (16 cases) was 4.46 ± 3.40 ($P < 0.005$), 4.14 ± 3.01 ($P < 0.001$) and 4.84 ± 3.56 ($P < 0.001$) respectively (Figure 1D, E and F), whereas in squamous cell carcinomas these ratios were 1.22 ± 0.99, 1.89 ± 1.71 and 1.83 ± 1.16 respectively (Figure 1D, E and F). Thus, the expression of cyclin D1, cdk4 and cdk6 in squamous cell carcinomas was not different from those in the adjacent non-cancerous regions. In contrast, the expression of cyclin D1, cdk4 and cdk6 was markedly increased in adenocarcinomas.

### Immunohistochemical detection of cyclin D1

The optimized protocol described in Materials and Methods was applied to a total of ten lung cancers, including five cases of adenocarcinomas and five with squamous cell carcinomas. Typical immunostaining pattern of cyclin D1 in adenocarcinoma and squamous cell carcinoma is shown in Figure 2. In adenocarcinoma with strong nuclear staining (arrows), the cytoplasm was also weakly stained. However, infiltrating inflammatory cells (lymphocytes) were not stained (Figure 2A). In squamous cell carcinoma, cyclin D1 was weakly stained in cancer cells (Figure 2B). Corresponding normal tissue did not show specific staining (Figure 2C). The results of the immunohistochemical study concurred with Western blot analysis.

### SDS-PAGE profile of GST-Rb fusion protein

GST-Rb fusion protein was used as the substrate to measure cyclin D1-related kinase activity. As shown in Figure 3A, staining of the GST-Rb fusion protein resolved by SDS-PAGE showed a single band at a molecular size of 46 kDa, as suggested by the previous report (Meyerson and Harlow, 1994). We confirmed that this band is a part of Rb protein by means of Western blot using Rb-specific monoclonal antibody (data not shown).

### Activation of cyclin D1-related kinase in lung cancer

Cyclin D1-related kinase activity was measured using in-gel kinase assay. Representative results are shown in Figure 3B. Cyclin D1-related kinase activity in lung cancer was significantly higher than that in adjacent non-cancerous regions (mean T/N ratio; 2.14 ± 2.01, $P < 0.05$). On the other hand, samples immunoprecipitated with non-immune control mouse IgG did not show any corresponding band. The mean T/N kinase activity ratio for adenocarcinoma was 3.05 ± 2.15 ($P < 0.05$), in contrast to 0.83 ± 0.67 for squamous cell carcinoma (Figure 4A). Thus, cyclin D1-related kinase activity was markedly increased in adenocarcinomas.

### Western blot of phosphorylated Rb

Rb protein in tumour tissues was more highly phosphorylated than those in adjacent non-cancerous region by Western blot using specific phospho-Rb polyclonal antibody (Figure 3C).

 *© 1999 Cancer Research Campaign*

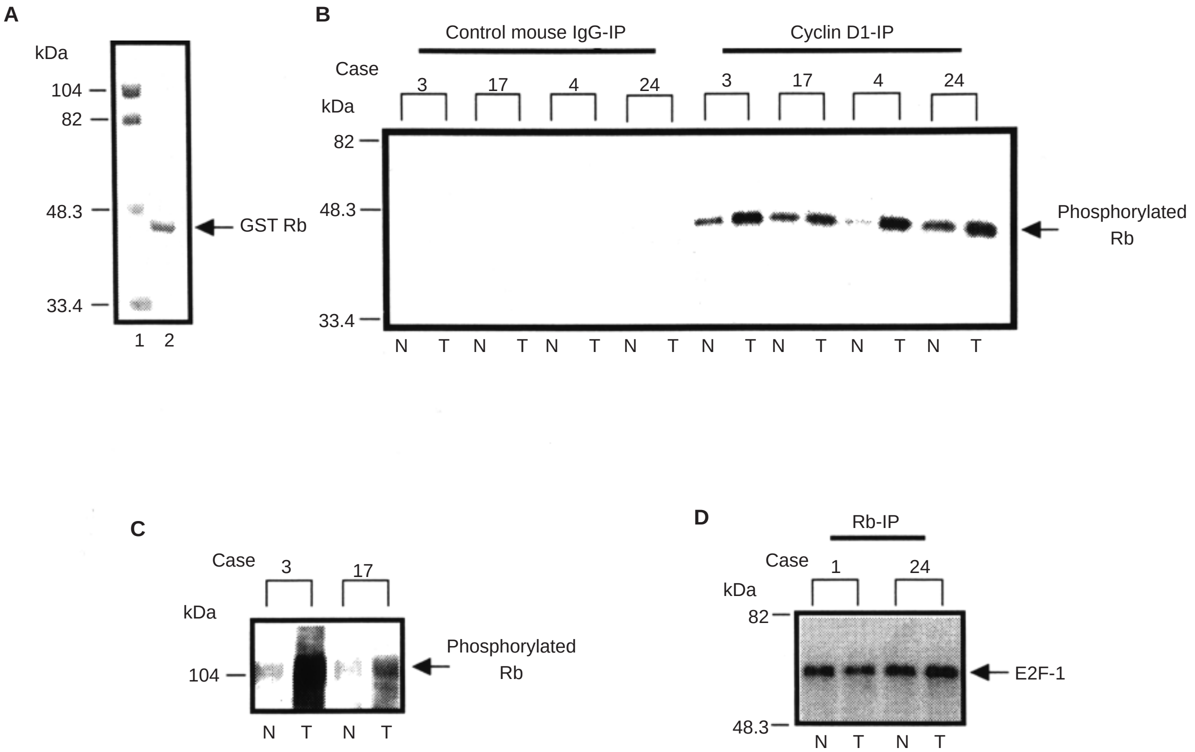

**Figure 3** (**A**) SDS-PAGE profile of GST-Rb fusion protein. Molecular weight marker (lane 1). Staining of the GST-Rb fusion protein resolved by SDS-PAGE showed a single band at the molecular size of 46 kDa (lane 2). (**B**) Cyclin D1-related kinase activity in non-tumourous (N) and tumourous (T) portions of lung cancer. Lysates containing 200 μg of total cellular protein were prepared as described in Materials and Methods. The protein was precipitated with excess cyclin D1 monoclonal antibody or non-immune control mouse IgG, incubated for 30 min with 1.0 μg GST-Rb fusion protein and 10 μCi of [$\gamma$-$^{32}$P] ATP in 20 μl of kinase buffer, then resolved on 12.5% SDS-polyacrylamide gels. Arrow indicates a band corresponding to phosphorylated Rb fusion protein. Cases 3 and 4, adenocarcinoma; cases 17 and 24, squamous cell carcinoma. (**C**) Western blot of phosphorylated pRb of amino acid residue corresponding to Ser780 in adenocarcinoma (case 3) and squamous cell carcinoma (case 17) tissue from the lung. Note that strong 105 kDa band of phosphorylated Rb was detected in the T tissues. (**D**) Western blot of Rb-bound E2F-1. Rb-bound E2F-1 was diminished in adenocarcinoma (case 1), but not in squamous cell carcinoma (case 24)

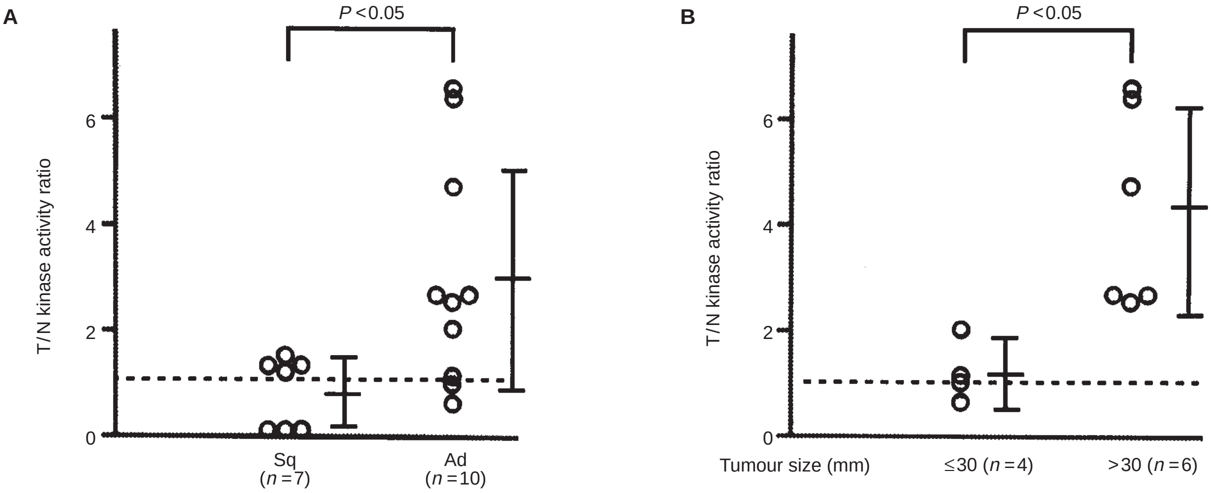

**Figure 4** (**A**) Comparison of the T/N ratio of cyclin D1-related kinase activity in adenocarcinomas (Ad) and squamous cell carcinomas (Sq). The T/N ratio of kinase activity for adenocarcinomas was significantly higher than that for squamous cell carcinomas (3.05 ± 2.15 vs 0.83 ± 0.67, $P < 0.05$). (**B**) Comparison of the T/N ratio of cyclin D1-related kinase activity for adenocarcinomas in two groups: tumour size ≤ 30 mm and tumour size > 30 mm. The difference between the two groups was significant ($P = 0.05$, Student's *t*-test). Values represent the mean ± s.d. of cyclin D1-related kinase activity for each group

### Western blot of Rb-bound E2F-1

Immunoprecipitates with anti-Rb were immunoblotted with anti-E2F-1. As shown in Figure 3D, Rb-bound E2F-1 was diminished in the tumor sample of adenocarcinoma, but not in squamous cell carcinoma.

### Relationship between cyclin D1 and its kinase activity

There was a significant positive correlation between the tumour versus non-tumourous counterpart ratio (T/N ratio) of the amount of cyclin D1 to cyclin D1-related kinase activity ($r = 0.76$, $P < 0.001$).

*© 1999 Cancer Research Campaign*

### Relationship between cyclin D1-related kinase activation and the clinicohistopathological characteristics of adenocarcinoma

The T/N ratio of cyclin D1-related kinase activity increased with the tumour size of adenocarcinomas ($r$ = 0.66, $P$ = 0.14). In the tumours with size > 30 mm, the T/N ratio of cyclin D1-related kinase activity (4.28 ± 1.90) was significantly higher ($P$ = 0.015) than those with size ≤ 30 mm (1.21 ± 0.59) (Figure 4B). However the T/N ratio of cyclin D1-related kinase activity was not correlated with advances in stage classification of adenocarcinomas ($r$ = –0.08, $P$ = 0.9). The T/N ratio of cyclin D1-related kinase activity was not significant for the degree of cell differentiation of adenocarcinomas, although there was an increased tendency in poorly differentiated adenocarcinoma.

On the other hand, the T/N ratio of cyclin D1-related kinase activity in squamous cell carcinomas was not associated with the size of cancer ($r$ = 0.09, $P$ = 0.59).

### DISCUSSION

The major finding in this study is that an increased cyclin D1-related kinase activity was found in approximately 70% of adenocarcinomas in lung cancer when compared with adjacent nontumorous tissues, whereas this kinase activity was not increased in squamous cell carcinomas. As shown in Figure 3C and D, phosphorylation of pRb was also detected on Western blot, and Rb-bound E2F-1 was decreased in adenocarcinoma, but not in squamous cell carcinoma. These data strongly support the result of in-gel kinase assay.

The cyclin D1 facilitates kinase activity by forming the complexes with its catalytic partners of cdk4 and cdk6, and cells leaving G0 progress through G1 to S phase. In the present study, we studied the amounts of cdk4 and cdk6 in lung cancer tissues. In most adenocarcinoma tissues, the amounts of cdk4 and cdk6, as well as cyclin D1, increased. These data suggest that the enhancement of cdk4 and cdk6 levels may be associated with the elevation of cyclin D1-related kinase activity in adenocarcinomas, and might be involved in the progress of malignancy in adenocarcinomas. On the other hand, neither cdk4 nor cdk6 levels increased in squamous cell carcinomas. These observations may suggest that the tumorigenesis of squamous cell carcinomas may depend on other cyclins or their catalytic partners. For instance, cyclin D2, E (Tremblay et al, 1992; Leach et al, 1993; Keyomarsi et al, 1994) and cyclin A (Wang et al, 1990) are also known to be involved in tumorigenesis.

The present findings are intriguing because the cyclin D1 gene amplification in adenocarcinomas has been rarely found in primary lung cancers (Berenson et al, 1990; Betticher et al, 1996). Instead, we have shown the enhancement of cyclin D1-related kinase activity and phosphorylation of pRb in lung cancer, especially in adenocarcinomas. These results suggest that the activation of cyclin D1-related kinase might play an important role in tumorigenesis of lung cancer.

The relationship between cyclin D1-related kinase activation and clinicopathological features of lung cancer was then evaluated. Cyclin D1-related kinase activity increased proportionally with tumour size, indicating that it was significantly higher in adenocarcinomas with diameters greater than 30 mm (Figure 4B). Therefore, the possibility that the activation of cyclin D1-related kinase is related to the development of adenocarcinomas cannot be denied.

Cyclin D1 is involved in tumour cell differentiation in a subset of lung cancers (Berenson et al, 1990; Betticher et al, 1996) Mate et al (1996) have reported that cyclin D1 overexpression in protein levels might play an important role in the process of tumour differentiation. In the present study, the distribution of cyclin D1 immunostaining in lung cancer seemed to be inversely related to the cytoplasmic differentiation, since poorly differentiated areas tended to yield a strong signal, whereas well-differentiated zones were devoid of immunoreaction (data not shown). In addition, cyclin D1-related kinase activity showed an increased tendency in moderately and poorly differentiated adenocarcinoma compared with well-differentiated, as shown in Table 1. These data suggest that the enhancement of cyclin D1-related kinase activity might play an important role in the tumour cell differentiation.

In conclusion, evidence suggests that activation of cyclin D1-related kinase has a significant role in the malignant transformation of lung tissues and is closely related to the progression of lung cancer, especially in adenocarcinomas. Thus, the suppression of cyclin D1-related kinase activity may provide a novel strategy in overcoming the development and invasion of adenocarcinomas. Further studies are necessary to investigate and evaluate these conclusions.

### ACKNOWLEDGEMENTS

This work was supported by a Grant-in-Aid for Scientific Research from the Ministry of Education, Science and Culture of Japan.

### REFERENCES

Baldin V, Lukas J, Marcote MJ, Pagano M and Draetta G (1993) Cyclin D1 is a nuclear protein required for cell cycle progression in G1. *Genes Dev* **7**: 812–821

Berenson JR, Koga H, Yang J, Pearl J, Holmes EC and Figlin R (1990) Frequent amplification of the *bcl*-1 locus in poorly differentiated squamous cell carcinoma of the lung. *Oncogene* **5**: 1343–1348

Betticher DC, Heighway J, Hasleton PS, Altermatt HJ, Ryder WDJ, Cerny T and Thatcher N (1996) Prognostic significance of CCND1 (cyclin D1) overexpression in primary resected non-small-cell lung cancer. *Br J Cancer* **73**: 294–300

Hall M and Peters G (1996) Genetic alterations of cyclins, cyclin-dependent kinases, and Cdk inhibitors in human cancer. *Adv Cancer Res* **68**: 67–108

Hsu SM, Raine L and Fanger H (1981) Use of avidin-biotin-peroxidase complex (ABC) in immunoperoxidase techniques: a comparison between ABC and unlabeled antibody (PAP) procedures. *J Histochem Cytochem* **29**: 577–580

Hunter T and Pines J (1994) Cyclins and cancer II: cyclin D and Cdk inhibitors come of age. *Cell* **79**: 573–582

Keyomarsi K, O'Leary N, Molnar G, Lees E, Fingert HJ and Pardee AB (1994) Cyclin E, a potential prognostic marker for breast cancer. *Cancer Res* **54**: 380–385

Kitagawa M, Higashi H, Jung HK, Takahashi I, Ikeda M, Tamai K, Kato J, Segawa K, Yoshida E, Nishimura S and Taya Y (1996) The consensus motif for phosphorylation by cyclin D1-Cdk4 is different from that for phosphorylation by cyclin A/E-Cdk2. *EMBO J* **15**: 7060–7069

Laemmli UK (1970) Cleavage of structural proteins during the assembly of the head of bacteriophage T4. *Nature* **227**: 680–685

Leach FS, Elledge SJ, Shell CJ, Willson JKV, Markowitz S, Kinzler KW and Vogelstein B (1993) Amplification of cyclin genes in colorectal carcinomas. *Cancer Res* **53**: 1986–1989

Mate JL, Ariza A, Aracil C, Lopez D, Isamat M, Perez-Piteira J and Navas-Palacios JJ (1996) Cyclin D1 overexpression in non-small cell lung carcinoma: correlation with Ki67 labelling index and poor cytoplasmic differentiation. *J Pathol* **180**: 395–399

© 1999 Cancer Research Campaign

Matsushime H, Roussel MF, Ashmun RA and Sherr CJ (1991) Colony-stimulating factor 1 regulates novel cyclins during the G1 phase of the cell cycle. *Cell* **65**: 701–713

Matsushime H, Ewen ME, Strom DK, Kato J, Hanks SK, Roussel MF and Sherr CJ (1992) Identification and properties of an atypical catalytic subunit ($p34^{PSK-J3}$/cdk4) for mammalian D type G1 cyclins. *Cell* **71**: 323–334

Meyerson M and Harlow E (1994) Identification of G1 kinase activity for cdk6, a novel cyclin D partner. *Mol Cell Biol* **14**: 2077–2086

Okayama H, Nagata A, Jinno S, Murakami H, Tanaka K and Nakashima N (1996) Cell cycle control in fission yeast and mammals: identification of new regulatory mechanisms. *Adv Cancer Res* **69**: 17–62

Quelle DE, Ashmun RA, Shurtleff SA, Kato J, Barsagi D, Roussel MF and Sherr CJ (1993) Overexpression of mouse D-type cyclins accelerates G1 phase in rodent fibroblasts. *Genes Dev* **7**: 1559–1571

Sherr CJ (1996) Cancer cell cycles. *Science* **274**: 1672–1677

Taya Y (1995) Cell cycle-dependent phosphorylation of the tumor suppressor RB protein. *Mol Cells* **5**: 191–195

Towbin H, Stadhelin T and Gordon J (1979) Electrophoretic transfer of protein from polyacrylamide gel to nitrocellulose sheets: procedure and some applications. *Proc Natl Acad Sci USA* **76**: 4350–4354

Tremblay PJ, Kozak CA and Jolicoeur P (1992) Identification of a novel gene, Vin-1, in murine leukemia virus-induced T-cell leukemias by provirus insertional mutagenesis. *J Virol* **66**: 1344–1353

Wang J, Chenivesse X, Henglein B and Brechot C (1990) Hepatitis B virus integration in a cyclin A gene in a hepatocellular carcinoma. *Nature* **343**: 555–557

*© 1999 Cancer Research Campaign*
